# Evaluating shared genetic influences on nonsyndromic cleft lip/palate and oropharyngeal neoplasms

**DOI:** 10.1002/gepi.22343

**Published:** 2020-07-24

**Authors:** Laurence J. Howe, Gibran Hemani, Corina Lesseur, Valérie Gaborieau, Kerstin U. Ludwig, Elisabeth Mangold, Paul Brennan, Andy R. Ness, Beate St Pourcain, George Davey Smith, Sarah J. Lewis

**Affiliations:** ^1^ Medical Research Council Integrative Epidemiology Unit, Population Health Sciences University of Bristol Bristol UK; ^2^ Institute of Cardiovascular Science University College London London UK; ^3^ Max Planck Institute for Psycholinguistics Nijmegen The Netherlands; ^4^ Section of Genetics International Agency for Research on Cancer Lyon France; ^5^ Institute of Human Genetics University of Bonn Bonn Germany; ^6^ NIHR Bristol Biomedical Research Centre University Hospitals Bristol Bristol UK; ^7^ Weston NHS Foundation Trust University of Bristol Bristol UK

**Keywords:** birth defects, genetic epidemiology, Mendelian randomization, oral cancers, orofacial clefts, polygenic risk scores

## Abstract

It has been hypothesised that nonsyndromic cleft lip/palate (nsCL/P) and cancer may share aetiological risk factors. Population studies have found inconsistent evidence for increased incidence of cancer in nsCL/P cases, but several genes (e.g., *CDH1*, *AXIN2*) have been implicated in the aetiologies of both phenotypes. We aimed to evaluate shared genetic aetiology between nsCL/P and oral cavity/oropharyngeal cancers (OC/OPC), which affect similar anatomical regions. Using a primary sample of 5,048 OC/OPC cases and 5,450 controls of European ancestry and a replication sample of 750 cases and 336,319 controls from UK Biobank, we estimate genetic overlap using nsCL/P polygenic risk scores (PRS) with Mendelian randomization analyses performed to evaluate potential causal mechanisms. In the primary sample, we found strong evidence for an association between a nsCL/P PRS and increased odds of OC/OPC (per standard deviation increase in score, odds ratio [OR]: 1.09; 95% confidence interval [CI]: 1.04, 1.13; *p* = .000053). Although confidence intervals overlapped with the primary estimate, we did not find confirmatory evidence of an association between the PRS and OC/OPC in UK Biobank (OR 1.02; 95% CI: 0.95, 1.10; *p* = .55). Mendelian randomization analyses provided evidence that major nsCL/P risk variants are unlikely to influence OC/OPC. Our findings suggest possible shared genetic influences on nsCL/P and OC/OPC.

## 1 INTRODUCTION

Nonsyndromic cleft lip/palate (nsCL/P) is a birth defect characterised by lack of fusion of structures in the upper lip and palate, with a complex aetiology influenced by both genetic and environmental factors (Dixon, Marazita, Beaty, & Murray, 2011; Mossey, Little, Munger, Dixon, & Shaw, 2009). Evidence from epidemiological population‐based studies that individuals with birth defects have increased incidence of cancer (Bjørge, Cnattingius, Lie, Tretli, & Engeland, 2008; Carozza, Langlois, Miller, & Canfield, 2012; Fisher et al., 2012; Johnson et al., 2017) suggests that birth defects, including nsCL/P, may have shared aetiology with cancer. However, for nsCL/P specifically, the evidence for increased incidence of cancers among cases and unaffected first degree relatives is largely inconsistent, with findings limited by available sample sizes (Bille et al., 2005; Bjørge et al., 2008; Carozza et al., 2012; Johnson et al., 2017; Vieira, Khaliq, & Lace, 2012; Zhu et al., 2002).

There are several practical limitations of comparing cancer incidence in nsCL/P cases with incidence in the general population. First, the co‐occurrence of nsCL/P and cancer is relatively modest because of the modest prevalence of nsCL/P (around 1 in 700; Mossey et al., 2009), and the two phenotypes typically differ in timing of onset. Second, cancers are highly heterogeneous across subtypes (e.g., childhood leukaemia and colorectal cancer), are highly distinct and stratifying by cancer subtype would further reduce statistical power. Third, syndromic and nonsyndromic forms of CL/P have different aetiologies and can be difficult to distinguish, suggesting that combining them together could be problematic if they have differences in cancer risk.

An alternative approach to evaluating the co‐occurrence of nsCL/P and cancer is to explore shared genetic risk factors. Previous studies have found several genes implicated in both nsCL/P and cancer (Dunkhase et al., 2016), notably *CDH1* (Hozyasz et al., 2014; Machado et al., 2017; Vogelaar et al., 2012) which is linked to gastric and breast cancer (Pharoah, Guilford, & Caldas, 2001), and *AXIN2* (Letra, Menezes, Granjeiro, & Vieira, 2009; Letra et al., 2012; Machado et al., 2017; Mostowska et al., 2012) which is associated with colorectal cancer and tooth agenesis (Liu et al., 2000; Lammi et al., 2004). These findings suggest that common biological pathways may influence nsCL/P and cancer risk, but the extent to which the two phenotypes share genetic risk factors is currently unclear.

Two methods for evaluating shared genetic risk factors between two traits are polygenic risk scores (PRS) and Mendelian randomization (Davey Smith & Ebrahim, 2003). PRS are scores consisting of multiple genetic variants associated with a phenotype that can be used to evaluate genetic overlap between two traits (Dudbridge, 2013). Similarly, Mendelian randomization uses genetic variants robustly associated with a trait, typically at genome‐wide significance, in an instrumental variable framework to evaluate possible causal relationships (Davey Smith & Hemani, 2014). In this instance, Mendelian randomization can be applied to test the possibility that common nsCL/P genetic variants, a latent measure of an individual's underlying liability to nsCL/P, influence cancer risk. Previous studies have demonstrated the utility of nsCL/P PRS and Mendelian randomization for disentangling mechanistic relationships (Dardani et al., 2020; Howe et al., 2018, 2019). For example, these approaches were used to provide evidence of shared genetic influences between nsCL/P and facial morphology (Howe et al., 2018).

A group of cancers that are strong candidates for shared genetic aetiology with nsCL/P are cancers of the oral cavity and oropharynx (OC/OPC) because of the similar anatomical sites affected. Major risk factors for OC/OPC include alcohol consumption, tobacco use and human papillomavirus infection (IARC Working Group on the Evaluation of Carcinogenic Risks to Humans, 2007) but OC/OPC also has a substantial heritable component (Lesseur et al., 2016). The possibility of shared genetic risk factors between nsCL/P and OC/OPC has not been previously investigated, possibly because of the relative rarity of both phenotypes.

Here, we first constructed nsCL/P PRS at a number of different thresholds using nsCL/P genome‐wide association study (GWAS) summary data. We then evaluated the association between the nsCL/P PRSs and OC/OPC using individual participant data on OC/OPC cases and controls using data from the largest OC/OPC GWAS (Lesseur et al., 2016) and UK Biobank. To differentiate between the two datasets, we refer to the samples as the International Agency for Research on Cancer (IARC) and UK Biobank samples, respectively. We then applied Mendelian randomization to both data sets to evaluate a potential causal effect of liability to nsCL/P, a latent measure of nsCL/P proxied by common nsCL/P variants, and OC/OPC. Finally, we used data from UK Biobank to explore associations between nsCL/P PRS and potential shared risk factors between nsCL/P and OC/OPC (alcohol/tobacco measures).

## 2 METHODS

### 2.1 Data sources

#### 2.1.1 Summary data from nsCL/P GWAS meta‐analysis

We used data from two nsCL/P GWAS, which have previously been meta‐analysed and published (Beaty et al., 2010; Ludwig et al., 2012; Mangold et al., 2010). The combined summary statistics were not publicly available, so meta‐analysis summary statistics were reconstructed by the authors, this has been described in detail previously (Howe et al., 2018, 2019). In brief, a Transmission Disequilibrium Test (TDT) was implemented in a genome wide association study of 638 parent‐offspring trios and 178 offspring duos of European descent (Beaty et al., 2010). The TDT results were then meta‐analysed with GWAS summary results on 399 cases and 1,318 controls from the Bonn‐II study, also of European descent (Mangold et al., 2010).

#### 2.1.2 IARC OC and OPC cancer GWAS data set

In this study, we used a data set of OC/OPC cases and controls which were part of a previous OC/OPC GWAS (Lesseur et al., 2016). In brief, the data set includes 6,034 OC, oropharyngeal or hypopharyngeal cancer cases and 6,585 controls. More information on the study design, genotyping, and phenotyping is contained in the Supporting Information Methods.

For analyses, the data set was restricted to 5,048 cases and 5,450 controls of recent European ancestry (confirmed by principal components analysis), which were split into two subsamples, based on the continent of the study centre (North America and Europe).

#### 2.1.3 UK Biobank

UK Biobank is a large‐scale cohort study of 502,655 participants aged between 40 and 69 years, who were recruited from 22 recruitment centres across the United Kingdom between 2006 and 2010. In UK Biobank, we identified OC/OPC cases and controls using secondary care data from Hospital Episode Statistics (HES) as well as the death and cancer registers. For secondary analyses, we used data on self‐reported alcohol consumption and tobacco smoking which were collected at baseline using a questionnaire. More information on the study design, genotyping and phenotyping is contained in the Supporting Information Methods.

For analyses, we used a subset of the study of 750 OC/OPC cases and 336,319 controls, after restricting to individuals of self‐reported “White British” descent and using kinship coefficients to remove individuals related to the greatest number of other individuals.

### 2.2 Statistical analyses

#### 2.2.1 The association of nsCL/P PRS with OC/OPC in the IARC sample

nsCL/P PRS were defined using the nsCL/P meta‐analysis GWAS summary statistics. The summary statistics were LD clumped (*r* ^2^ < .1 and 250 kb) at 11 different *p* value inclusion thresholds (.000001, .000005, .00001, 0.00004, .0001, .0005, .001, .005, .01, .05, .1) to generate sets of independent (in terms of LD) variants in each score. The PRS was then constructed using effect alleles and weightings based on the magnitude of effect on nsCL/P of each genetic variant taken from the meta‐analysis GWAS summary statistics. LD clumping was performed using PLINK (Purcell et al., 2007) with the 1000 Genomes (Phase 3; Genomes Project Consortium, 2015) CEU samples used as the reference panel.

Next, the 11 different nsCL/P PRS were constructed separately in the European and North American OC/OPC case–control sample using PLINK (Purcell et al., 2007) with an individual's PRS defined as the sum of weights across all variants in the PRS of the nsCL/P multiplied by the number of alleles. The associations between the nsCL/P PRS and OC/OPC case–control status were then estimated in the two subsamples using logistic regression adjusting for the first 10 genetic principal components, sex and age. Analyses were run separately for; all cases, OC cases only and oropharyngeal cases only (OPC). As a sensitivity analysis, we additionally removed cases and controls with less than 70% CEU ancestry. The effect sizes, standard errors and *p* values from the European and North American subsamples were meta‐analysed using a fixed‐effects model.

#### 2.2.2 The association of nsCL/P PRS with OC/OPC in UK Biobank

The UK Biobank data set was used as a follow‐up data set for analyses in the OC/OPC GWAS data set. For the analyses in UK Biobank, we used the nsCL/P PRS most strongly associated with OC/OPC in the OC/OPC GWAS data set, tested the association of this PRS with OC/OPC. The limited number of OC/OPC cases (*N* = 750) in our sample meant that it was not possible to stratify by OC/OPC subtype, so all cases were analysed together. All analyses were adjusted for the first 10 genetic principal components, age, and sex.

#### 2.2.3 Mendelian randomization analysis of liability to nsCL/P on OC/OPC

Mendelian randomization was then applied to evaluate a causal relationship between liability to nsCL/P and OC/OPC. As genetic instruments for liability to nsCL/P, we used six genome‐wide significant single nucleotide polymorphisms (SNPs), as in a previous study (Howe et al., 2018; Ludwig et al., 2012; Table S2). Chosen variants have large effects on nsCL/P risk (Ludwig et al., 2012) suggesting that they can be utilised as effective genetic instruments. Although recent nsCL/P GWAS have identified additional genome‐wide significant markers in European populations (Leslie et al., 2017; Ludwig et al., 2016), we were limited by available summary data. SNP data for liability to nsCL/P were extracted from the nsCL/P meta‐analysis GWAS summary statistics.

SNP data for OC/OPC were extracted from a meta‐analysis of the IARC GWAS sample (European and North American subsamples) and UK Biobank (Lesseur et al., 2016). The GWAS in the IARC sample has been previously described in detail (Lesseur et al., 2016). In UK Biobank, the GWAS of 750 cases and 336,319 controls was conducted using logistic regression in PLINK v2.00 (Purcell et al., 2007). Sex, age, and the first 10 principal components were fitted as covariates in the model. The GWAS summary data from all data sets were meta‐analysed using METAL (Willer, Li, & Abecasis, 2010) to generate combined summary statistics for OC/OPC.

We then used the TwoSampleMR (Hemani et al., 2018) package in R to conduct Mendelian randomization analyses; reporting the inverse‐variance weighted estimate as the primary analysis. We also used several sensitivity analyses; MR Egger, weighted median, weighted mode and Cochran's *Q* heterogeneity test (Hemani, Bowden, & Davey Smith, 2018).

#### 2.2.4 nsCL/P PRS, alcohol consumption, and tobacco smoking

We tested the nsCL/P PRS (including SNPs with *p* < 0.1) for association with alcoholic units consumed per week and tobacco smoking pack years. All analyses were adjusted for sex, age and the first 10 genetic principal components.

## 3 RESULTS

### 3.1 nsCL/P PRS and risk of OC/OPC in the IARC sample

**Figure 1:**
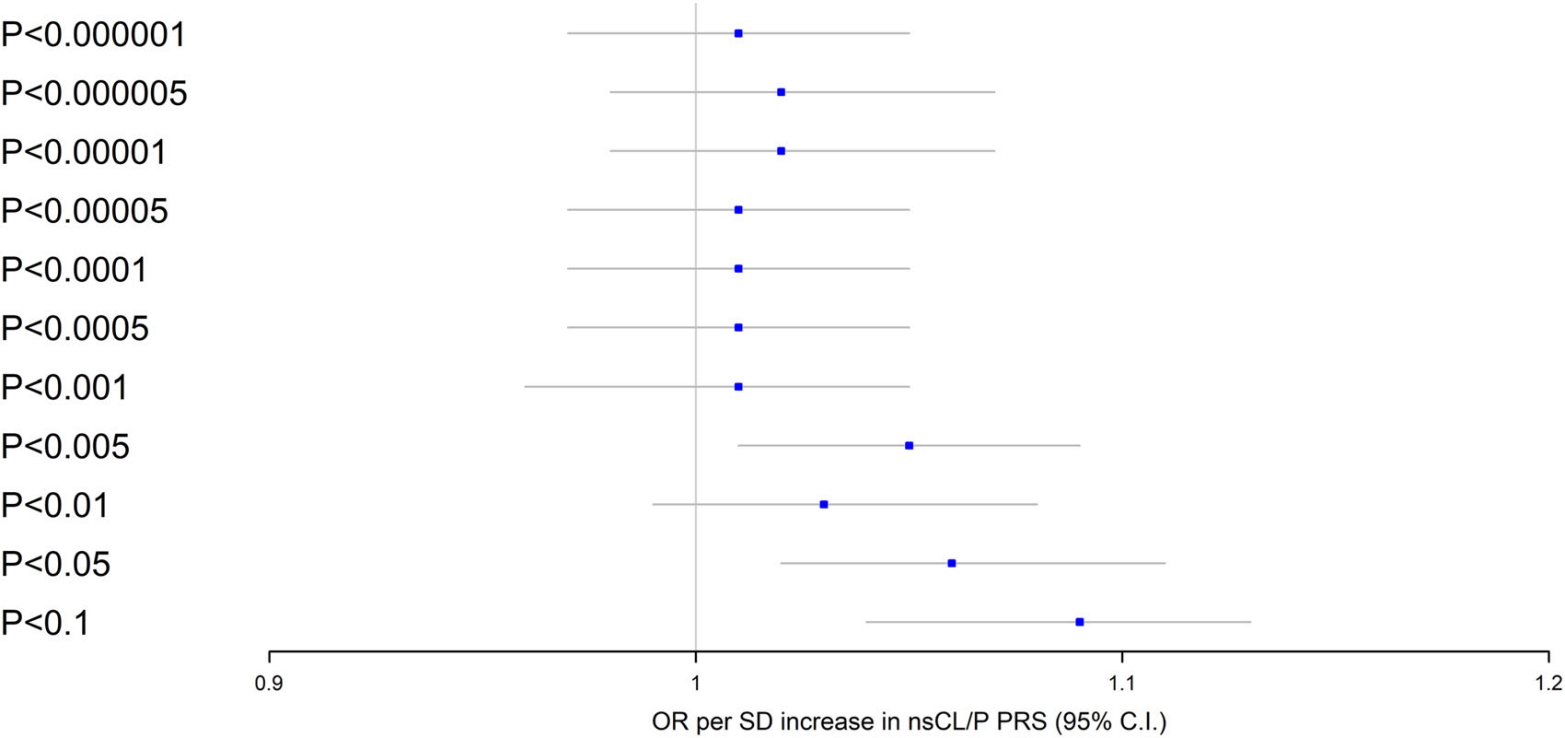
Association of nsCL/P PRS with OC/OPC in the International Agency for Research on Cancer sample. A forest plot showing the associations between different nsCL/P PRS, ranging from 10 SNPs to over 10,000 SNPs, and OC/OPC risk. nsCL/P, nonsyndromic cleft lip/palate; OC, oral cavity; OPC, oropharynx; PRS, polygenic risk score; SNP, single nucleotide polymorphism

We found strong evidence for an association between nsCL/P PRS and increased risk of OC/OPC in the IARC sample of 5,048 cases and 5,450 controls. The strongest association was for the nsCL/P PRS including SNPs with *p* < .1, where a 1 standard deviation (*SD*) increase in nsCL/P PRS was associated with increased odds of PC/OPC (odds ratio [OR]: 1.09; 95% confidence interval [CI]: 1.04, 1.13; *p* = .000053). PRS with more liberal inclusion thresholds (e.g., *p* < .05 and *p* < .1), which included thousands of SNPs, were more strongly associated with risk of OC/OPC than more conservative inclusion thresholds (Figure 1 and Table 1).

**Table 1:** nsCL/P PRS and risk of OC/OPC

Polygenic risk score *p* value inclusion threshold	Number of SNPs in PRS	All OC/OPC subtypes against controls (5,048 cases and 5,450 controls)	
		OR (95% CI) per 1 *SD* increase in PRS	*p* Value
.000001	10	1.01 (0.97, 1.05)	.64
.000005	15	1.02 (0.98, 1.07)	.27
.00001	18	1.02 (0.98, 1.07)	.27
.00005	48	1.01 (0.97, 1.05)	.71
.0001	78	1.01 (0.97, 1.05)	.58
.0005	238	1.01 (0.97, 1.05)	.71
.001	424	1.01 (0.96, 1.05)	.79
.005	1,607	1.05 (1.01, 1.09)	.021
.01	2,777	1.03 (0.99, 1.08)	.10
.05	8,620	1.06 (1.02, 1.11)	.0026
.1	12,614	1.09 (1.04, 1.13)	.000053

Abbreviations: CI, confidence interval; nsCL/P, nonsyndromic cleft lip/palate; OC, oral cavity; OPC, oropharynx; PRS, polygenic risk score; *SD*, standard deviation; SNP, single nucleotide polymorphism.

Similarly, there was strong evidence for an association between the nsCL/P PRS and the primary OC/OPC subtypes, oropharyngeal and OC cancer. A 1 *SD* increase in nsCL/P PRS (*p* < 0.1) was associated with increased odds of both oropharyngeal cancer (OR: 1.10; 95% CI: 1.04, 1.16; *p* = .00079) and OC cancer (OR: 1.12; 95% CI: 1.06, 1.17; *p* = .000016; Table S1).

### 3.2 nsCL/P PRS and risk of OC/OPC in UK Biobank

In the UK Biobank sample of 750 cases and 336,319 controls, we attempted to replicate the association between the nsCL/P PRS most strongly associated with OC/OPC in the IARC sample (*p* value inclusion threshold < .1). Here, we found no clear evidence that this PRS is associated with increased risk of OC/OPC (OR: 1.02; 95% CI: 0.95, 1.10; *p* = .55). However, confidence intervals overlapped with the IARC sample estimate (OR: 1.09 95% CI: 1.04, 1.13) and the meta‐analysis of the two estimates suggested evidence for an association (OR: 1.07; 95% CI: 1.03, 1.11; *p* = .0009).

### 3.3 Mendelian randomization: Liability to nsCL/P and risk of OC/OPC

Using combined GWAS summary data from the IARC and UK Biobank samples, 5,798 cases and 341,769 controls, we found little evidence for an effect of liability to nsCL/P on OC/OPC; a 1‐unit log odd increase in liability to nsCL/P was not strongly associated with OC/OPC (MR IVW: OR: 0.99; 95% CI: 0.95, 1.05; *p* = .84). Mendelian randomization sensitivity analyses generated consistent effect estimates (Table 2).

**Table 2:** Mendelian randomization analysis of liability to nsCL/P on OC/OPC risk

Test	Interpretation	OR (95% CI)	*p* Value
Inverse variance weighted	Primary result^a^	0.99 (0.95, 1.05)	.84
Heterogeneity of inverse variance weighted	Balanced pleiotropy	N/A	.59
MR‐Egger	Intercept test for directional pleiotropy^b^	0.002 (−0.058, 0.063)	.94
	Regression estimate^a^	0.99 (0.87, 1.13)	.69
Weighted median	Consistency^a^	0.99 (0.93, 1.06)	.77
Weighted mode	Consistency^a^	0.99 (0.92, 1.06)	.71

Abbreviations: CI, confidence interval; nsCL/P, nonsyndromic cleft lip/palate; OC, oral cavity; OPC, oropharynx; OR, odds ratio.

a Units: odds ratio for OC/OPC perper 1‐unit log odd increase in liability to nsCL/PnsCL/P.

b Units: average pleiotropic effect of a nsCL/P genetic variant on odds of OC/OPC.

### 3.4 nsCL/P PRS, alcohol consumption and tobacco smoking

To evaluate whether shared genetic effects could relate to interactions of the nsCL/P PRS with shared environmental risk factors we investigated the genetic overlap between the PRS and measures of alcohol consumption and tobacco smoking in UK Biobank. Again, we used the nsCL/P PRS most strongly associated with OC/OPC in the IARC sample (*p* < 0.1). We did not find strong evidence that the nsCL/P PRS was associated with any of the measures tested. A 1 *SD* increase in nsCL/P PRS corresponded to a −0.02 pack year decrease in lifetime smoking (95% CI: −0.07, 0.03; *p* = .46; *N* = 284,144) and a −0.05 units per week decrease in alcohol use (95% CI: −0.11, 0.01; *p* = .11; *N* = 336,026).

## 4 DISCUSSION

In this study, we found some evidence to suggest that nsCL/P PRS are associated with modestly increased risk of OC/OPC. Follow‐up Mendelian randomization analyses using well established nsCL/P risk SNPs as a latent measure of nsCL/P suggested no consistent association between key common nsCL/P variants and OC/OPC. These findings are consistent with our initial hypothesis that nsCL/P and OC/OPC share some genetic risk factors as opposed to the possibility of a causal relationship, which has previously been demonstrated for nsCL/P and philtrum width (Howe et al., 2018).

The specific shared genetic influences and relevant biological pathways that may have induced the association between the nsCL/P PRS and OC/OPC are currently unclear. While the Mendelian randomization approach includes only replicated variants with strong associations, the PRS approach is much more liberal and will include many variants with weaker associations. The increased variation explained by the PRS approach comes with the caveat that many variants of small effects will be included which have less clear direct relevant to nsCL/P. The interpretation of what a PRS is proxying for becomes increasingly complex as the number of SNPs included in the score increases. Indeed, the nsCL/P PRS most strongly associated with OC/OPC included over 10,000 SNPs. One possibility is that the genetic overlap may be attributable to certain genes being involved both in early development and tumour suppression. For example, the *CDH1* gene, thought to be related to nsCL/P and cancer subtypes, has been shown to be related to both axonal growth and patterning in the developing murine brain (Konishi, Stegmüller, Matsuda, Bonni, & Bonni, 2004), and tumour suppression (Berx, Becker, Höfler, & Van Roy, 1998).

Another possibility is that the nsCL/P PRS may capture environmental influences relating to both nsCL/P and cancer risk. Maternal behaviour such as cigarette smoking and alcohol consumption have been hypothesised to influence nsCL/P risk and both are established risk factors for OC/OPC. Given that the maternal genotype is correlated with the offspring genotype, it is plausible that nsCL/P PRS could capture effects of maternal environmental factors. The foetal genotype may also play an important role in the effect of environmental exposures on risk of orofacial clefts. For example, there is some evidence that the maternal and foetal *ADH1C* haplotype may modify the association between maternal alcohol consumption and risk of orofacial clefts via alcohol metabolism (Boyles et al., 2010). We did not find strong evidence that nsCL/P PRS are associated with alcohol and cigarette use in UK Biobank but were unable to test pathways relating to metabolism.

The findings of this study are consistent with previous genetic studies that have found evidence of loci associated with both nsCL/P and various cancer subtypes (Dunkhase et al., 2016; Hozyasz et al., 2014; Letra et al., 2009, 2012; Machado et al., 2017; Mostowska et al., 2012; Vogelaar et al., 2012). Our results are also consistent with a previous study exploring adult‐onset cancers in nsCL/P cases, which was underpowered to detect a modest effect (Bille et al., 2005).

This study is the first to investigate the genetic overlap between nsCL/P and OC/OPC. Previous epidemiological and genetic studies have explored the relationship between nsCL/P and all cancers, however, cancers arising from different organs may be aetiologically heterogeneous. Although similar results were found in analyses for the OC and OPC subtype analyses with these subtypes also aetiologically heterogeneous. In Western populations, smoking and alcohol are established risk factors for both OC and OPC, while HPV status is thought to be a risk factor for OPC only (Lesseur et al., 2016). Future work could evaluate genetic overlap between nsCL/P and other cancer subtypes to determine if the association is consistent across other cancer subtypes or localised specifically to oral tissues.

The use of a PRS as a genetic proxy for nsCL/P in our analyses is a considerable strength. PRS analyses have advantages over candidate gene or candidate SNP approaches used in previous studies (Dunkhase et al., 2016; Hozyasz et al., 2014; Letra et al., 2009, 2012; Machado et al., 2017; Mostowska et al., 2012; Vogelaar et al., 2012) because of the reduction in the number of statistical tests, and the potential to extend the evidence of genetic overlap at specific loci to examine genome‐wide genetic overlap. Although the nsCL/P GWAS data set was modestly sized, the construction of nsCL/P PRS in a much larger OC/OPC GWAS data set meant that analyses were well‐powered to detect a modest genetic overlap.

Nevertheless, there are several limitations of this study. First, the lack of a convincing replication of the PRS analysis in UK Biobank weakens the argument for genetic overlap between the two phenotypes. However, this may be because of the modest number of OC/OPC cases in the UK Biobank, with confidence intervals overlapping between the two studies. Second, the OC/OPC GWAS data set was highly ancestrally heterogeneous and included samples from 12 different epidemiological studies, including a case‐only study (the Head and Neck 5000). Therefore, it is possible that allele frequency differences between cases and controls relating to population differences could result in spurious associations with the nsCL/P PRS, although this is unlikely given the number of SNPs in the PRS. Third, with insufficient genetic instruments for OC/OPC, we did not perform a bidirectional Mendelian randomization analysis so cannot rule out the possibility that OC/OPC variants consistently affect nsCL/P risk. Fourth, we were unable to run stratified analysis investigating the possibility of heterogeneity between HPV and non‐HPV driven tumours. Fifth, in this study we treated nsCL/P subtypes (cleft lip with cleft palate, cleft lip only) homogeneously despite growing evidence that they are aetiologically distinct (Leslie et al., 2017; Ludwig et al., 2016; Sharp et al., 2017). This complicates the interpretation of the results because the OPC includes the soft palate while the OC includes the lips and hard palate. It is therefore, possible that the different nsCL/P subtypes may have different mechanistic relationships with the OC/OPC subtypes. Finally, we were unable to explore the effects of somatic mutations in the oral tissues which could influence both orofacial cleft risk and localized cancer.

To conclude, we found some evidence of shared genetic influences on nsCL/P and OC/OPC unrelated to alcohol or tobacco intake. Follow‐up analyses, potentially using additional datasets such as the Cleft Collective (www.bristol.ac.uk/dental/cleft-collective/), are required to investigate the possible common biological pathways between nsCL/P and OC/OPC, and to evaluate possible mechanistic relationships between nsCL/P and cancer subtypes affecting distinct areas to orofacial clefts.

## AUTHOR CONTRIBUTIONS

L. J. H., A. R. N., and S. J. L. conceptualised and designed the project. L. J. H. and C. L. performed statistical analyses. A. R. N., E. M., K. L., V. G., and P. B. were involved in data acquisition. L. J. H. drafted the manuscript and interpreted results under the supervision of G. H., G. D. S., B. S. P., and S. J. L. All authors contributed to critically revising the manuscript. All authors gave their final approval and agree to be accountable for all aspects of the work. All authors declare no relevant conflicts of interest to the publication of this manuscript.

## Supporting information

Supporting information

## Data Availability

Research Data are not shareThe nsCL/P family data set used to construct summary data described in this manuscript were obtained from dbGaP at https://www.ncbi.nlm.nih.gov/projects/gap/cgi-bin/study.cgi?study_id=phs000094.v1.p1 through dbGaP accession number (phs000094.v1.p1). Individuals interested in accessing summary data from the nsCL/P Bonn GWAS are encouraged to contact Elisabeth Mangold (e.mangold@uni‐bonn.de) or Kerstin Ludwig (kerstin.ludwig@uni‐bonn.de). The OC/OPC GWAS data is available from dbGaP at https://www.ncbi.nlm.nih.gov/projects/gap/cgi-bin/study.cgi?study_id=phs001202.v1.p1 through dbGaP accession number (phs001202.v1.p1). Summary data from the UK Biobank GWAS of OC/OPC, described in this manuscript, will be made available in the MR Base platform and the GWAS catalogue before publication.
