# Supplementary material for: Evaluating shared genetic influences on nonsyndromic cleft lip/palate and oropharyngeal neoplasms
Source: Genet Epidemiol. 2020 Jul 24;44(8):924–33. doi: 10.1002/gepi.22343 (PMC8240308; doi:10.1002/gepi.22343)
Supplement: Supplementary file 1 — Supporting information [file GEPI-44-924-s001.docx]

# Supplemental Methods

## IARC oral cavity and oropharyngeal cancer GWAS dataset

### Study description

In this study, we used a dataset of OC/OPC cases and controls which were part of an OC/OPC GWAS (Lesseur et al. 2016). Study participants were recruited from 12 epidemiological studies, mostly hospital-based case-control samples, based in North America, South America and Europe. This published GWAS included participants from: the European Prospective Investigation into Cancer and Nutrition (EPIC), the Head and Neck 5000 (HN5000), the Alcohol-Related Cancer and Genetic Susceptibility Study in Europe (ARCAGE), the International Agency for Research on Cancer (IARC) Central Europe Study, the IARC Oral Cancer Study, the Maastricht Study, the Rome Study, the Carolina Head and Neck cancer study, the University of Pittsburgh Head and Neck Cancer Study, the Toronto Study, the IARC Latin America Cancer Study and the Head and Neck Genome Project (GENCAPO). For the purposes of analyses in this study, the dataset was restricted to 5,048 cases and 5,450 controls of recent European ancestry (confirmed by principal components analysis), which were split into two subsamples, based on the continent of the study centre (North America and Europe).

### Phenotyping

The OC/OPC subtypes (oral cavity, oropharyngeal, hypopharyngeal and overlapping at multiple sites) were identified using the following ICD 10 codes from hospitalisation events: oral cavity cancer (C02.0–C02.9, C03.0–C03.9, C04.0–C04.9, C05.0–C06.9), oropharyngeal (C01, C02.4, C09.0–C10.9), hypopharyngeal (C13.0–C13.9) and overlapping; characterised by a neoplasm across two or more of the lip, oral cavity or pharynx (C14) (Lesseur et al. 2016).

### Genotyping and imputation

Genotyping and quality control has been described in detail previously (Lesseur et al. 2016). In brief, DNA samples from blood or buccal cells were genotyped using the Illumina OncoArray, a custom cancer array. PLINK (Purcell et al. 2007) was first used to exclude samples and SNPs with excessively high missingness (>20%). After the initial exclusions, in a second round of quality control, samples and SNPs with missingness (>5%) were removed. Samples with chromosomal errors, excessive or minimal autosomal heterozygosity, unexpected relatedness (IBD > 0.3) and expected experimental duplicate pairs were also removed. The dataset was divided into the three geographic regions of the study centres (North America, South America and Europe), and SNPs deviating from HWE (P<1x10^-7^) as well as population outliers were removed. The directly genotyped data were then imputed using the Michigan Imputation Server with SHAPEIT ^(Delaneau et al. 2013)^ used for pre-phasing, Minimac3 (Das et al. 2016) used for the genotype imputation and the Haplotype Reference Consortium panel ^(McCarthy et al. 2016)^ used as the reference panel.

## UK Biobank

### Study description

UK Biobank is a large-scale cohort study of 502,655 participants aged between 40-69 years, who were recruited from 22 recruitment centres across the United Kingdom between 2006 and 2010. The majority of the cohort have been genotyped and phenotype data for study participants has been collected using a questionnaire completed at baseline and also via linkage with hospital data and cancer registries (Sudlow et al. 2015).

### Phenotyping

OC/OPC cases were identified using Hospital Episode Statistics (HES), the death register (primary cause of death) and the cancer registry. The following ICD10 codes from any source were used to define cases: oral cavity cancer (C02.0–C02.9, C03.0–C03.9, C04.0–C04.9, C05.0–C06.9), oropharyngeal (C01, C02.4, C09.0–C10.9), hypopharyngeal (C13.0–C13.9) and overlapping (C14).

Self-reported alcohol consumption data were collected at baseline using a questionnaire. Participants were asked for their alcohol drinking status (current, former, never) and for estimates of their average weekly intake of a range of different alcoholic beverages (red wine, white wine, champagne, beer, cider, spirits, fortified wine). From these variables, an average intake of alcoholic units per week was derived by summing the estimated intakes of the different alcoholic beverages consumptions across the seven drink types, as in a previous study (Clarke et al. 2017). Individuals reporting their current intake frequency of “one to three times a month”, “special occasions only” or “never” were assumed to have a weekly alcohol consumption volume of 0.

Similarly, self-reported tobacco smoking data were collected at baseline using a questionnaire. Participants were asked their tobacco smoking status; current, former, never. Current smokers were asked to estimate the number of cigarettes smoked per day and the age they started smoking which was used to generate a pack years measure. Former smokers were asked to estimate the number of cigarettes smoked per day previously, the age they started smoking and the age they stopped smoking, to generate a pack years measure. Individuals reporting their tobacco smoking status as “never smokers” were assumed to have tobacco pack years of 0. For the purposes of our analyses, individuals with alcoholic consumption volume or tobacco pack years more than five standard deviations away from the mean were removed from relevant analyses.

### Genotyping and imputation

UK Biobank participants (n=488,377) were assayed using two similar genotyping arrays, the UK BiLEVE Axiom™ Array by Affymetrix1 (N= 49,950) and the closely related UK Biobank Axiom™ Array (N= 438,427). Directly genotyped variants were pre-phased using SHAPEIT3 (O'Connell et al. 2016) and then imputed using Impute4 using the UK10K (Consortium 2015), Haplotype Reference Consortium (McCarthy et al. 2016) and 1000 Genomes Phase 3 (Genomes Project Consortium 2015) reference panels. Post-imputation, data were available on approximately ~96 million genetic variants (Allen et al. 2014; Bycroft et al. 2017). We used a subset of the study of 750 OC/OPC cases and 336,319 controls, after restricting to individuals of self-reported “White British” descent and using kinship coefficients to remove individuals related to the greatest number of other individuals (Mitchell et al. 2017).

## References

Allen NE, Sudlow C, Peakman T, Collins R. 2014. Uk biobank data: Come and get it. American Association for the Advancement of Science.

Bycroft C, Freeman C, Petkova D, Band G, Elliott LT, Sharp K, Motyer A, Vukcevic D, Delaneau O, O'Connell J. 2017. Genome-wide genetic data on~ 500,000 uk biobank participants. bioRxiv.166298.

Clarke T-K, Adams MJ, Davies G, Howard DM, Hall LS, Padmanabhan S, Murray AD, Smith BH, Campbell A, Hayward C. 2017. Genome-wide association study of alcohol consumption and genetic overlap with other health-related traits in uk biobank (n= 112 117). Molecular Psychiatry. 22(10):1376.

Consortium UK. 2015. The uk10k project identifies rare variants in health and disease. Nature. 526(7571):82-90.

Das S, Forer L, Schönherr S, Sidore C, Locke AE, Kwong A, Vrieze SI, Chew EY, Levy S, McGue M. 2016. Next-generation genotype imputation service and methods. Nature Genetics. 48(10):1284.

Delaneau O, Zagury J-F, Marchini J. 2013. Improved whole-chromosome phasing for disease and population genetic studies. Nature Methods. 10(1):5-6.

Genomes Project Consortium. 2015. A global reference for human genetic variation. Nature. 526(7571):68-74.

Lesseur C, Diergaarde B, Olshan AF, Wünsch-Filho V, Ness AR, Liu G, Lacko M, Eluf-Neto J, Franceschi S, Lagiou P. 2016. Genome-wide association analyses identify new susceptibility loci for oral cavity and pharyngeal cancer. Nature Genetics. 48(12):1544.

McCarthy S, Das S, Kretzschmar W, Delaneau O, Wood AR, Teumer A, Kang HM, Fuchsberger C, Danecek P, Sharp K. 2016. A reference panel of 64,976 haplotypes for genotype imputation. Nature Genetics. 48(10):1279.

Uk biobank genetic data: Mrc-ieu quality control, version 1, 13/11/2017. 2017. [accessed].

O'Connell J, Sharp K, Shrine N, Wain L, Hall I, Tobin M, Zagury J-F, Delaneau O, Marchini J. 2016. Haplotype estimation for biobank-scale data sets. Nature Genetics. 48(7):817-820.

Purcell S, Neale B, Todd-Brown K, Thomas L, Ferreira MA, Bender D, Maller J, Sklar P, De Bakker PI, Daly MJ. 2007. Plink: A tool set for whole-genome association and population-based linkage analyses. The American Journal of Human Genetics. 81(3):559-575.

Sudlow C, Gallacher J, Allen N, Beral V, Burton P, Danesh J, Downey P, Elliott P, Green J, Landray M. 2015. Uk biobank: An open access resource for identifying the causes of a wide range of complex diseases of middle and old age. PLoS Medicine. 12(3):e1001779.

Supplemental Table 1: Association of nsCL/P PRS with OC/OPC subtypes

| Polygenic risk score P-value inclusion threshold | Oropharyngeal cases only  (2297 cases and 5182 controls) | | Oral cavity cases only  (2463 cases and 5182 controls) | |
| --- | --- | --- | --- | --- |
|  | OR (95% C.I.)  Per 1 S.D. increase in PRS | P | OR (95% C.I.)  Per 1 S.D. increase in PRS | P |
| 0.000001 | 1.03 (0.97, 1.08) | 0.37 | 1.01 (0.96, 1.07) | 0.58 |
| 0.000005 | 1.04 (0.98, 1.09) | 0.19 | 1.02 (0.97, 1.07) | 0.54 |
| 0.00001 | 1.03 (0.98, 1.09) | 0.25 | 1.02 (0.97, 1.07) | 0.44 |
| 0.00005 | 1.02 (0.96, 1.07) | 0.51 | 1.02 (0.97, 1.08) | 0.35 |
| 0.0001 | 1.03 (0.97, 1.08) | 0.38 | 1.03 (0.98, 1.09) | 0.20 |
| 0.0005 | 1.01 (0.96, 1.07) | 0.62 | 1.03 (0.98, 1.08) | 0.23 |
| 0.001 | 1.02 (0.97, 1.08) | 0.45 | 1.02 (0.97, 1.08) | 0.35 |
| 0.005 | 1.04 (0.99, 1.10) | 0.11 | 1.07 (1.02, 1.13) | 0.0061 |
| 0.01 | 1.04 (0.99, 1.10) | 0.12 | 1.05 (1.00, 1.10) | 0.073 |
| 0.05 | 1.07 (1.02, 1.13) | 0.011 | 1.09 (1.04, 1.15) | 0.00053 |
| 0.1 | 1.10 (1.04, 1.16) | 0.00079 | 1.12 (1.06, 1.17) | 0.000016 |

**Supplemental Table 2:** Mendelian randomization: nsCL/P SNPs

| **SNP** | **CHR:BP^1^** | **Effect Allele / Other Allele** | **nsCL/P Beta** | **nsCL/P S.E.** | **Primary sample** | | **UK Biobank** | |
| --- | --- | --- | --- | --- | --- | --- | --- | --- |
|  |  |  |  |  | **OC/OPC Beta** | **OC/OPC S.E.** | **OC/OPC Beta** | **OC/OPC S.E.** |
| rs7590268 | 2:43540125 | T/G | -0.33 | 0.065 | -0.048 | 0.035 | 0.106 | 0.062 |
| rs987525 | 8:129946154 | A/C | 0.83 | 0.091 | -0.039 | 0.035 | 0.079 | 0.062 |
| rs7078160 | 10:118827560 | A/G | 0.40 | 0.070 | 0.030 | 0.040 | 0.100 | 0.068 |
| rs8001641 | 13:80692811 | A/G | 0.36 | 0.058 | -0.026 | 0.030 | -0.027 | 0.052 |
| rs1873147 | 15:63312632 | A/G | -0.35 | 0.062 | -0.023 | 0.033 | 0.042 | 0.059 |
| rs227731 | 17:54773238 | T/G | -0.31 | 0.056 | 0.048 | 0.032 | -0.060 | 0.052 |

^1 CHR:BP – Chromosome and Base Pair Position on HG19^
